# CD133/prominin-1 is a potential therapeutic target for antibody-drug conjugates in hepatocellular and gastric cancers

**DOI:** 10.1038/sj.bjc.6604437

**Published:** 2008-06-10

**Authors:** L M Smith, A Nesterova, M C Ryan, S Duniho, M Jonas, M Anderson, R F Zabinski, M K Sutherland, H-P Gerber, K L Van Orden, P A Moore, S M Ruben, P J Carter

**Affiliations:** 1 Seattle Genetics Inc., 21823 30th Drive Southeast, Bothell, WA 98021, USA; 2 Celera, 45 West Gude Drive, Rockville, MD 20850, USA

**Keywords:** CD133, prominin-1, gastric, hepatocellular, antibody-drug conjugate

## Abstract

CD133/prominin-1 is a pentaspan transmembrane glycoprotein overexpressed in various solid tumours including colorectal and glioblastomas. CD133 was found here to be highly expressed in ⩾50% of pancreatic, gastric and intrahepatic cholangiocarcinomas. Quantitative flow cytometric analysis showed that a panel of established hepatocellular, pancreatic and gastric cancer cell lines expressed CD133 at levels higher than normal epithelial cells or bone marrow progenitor cells. A murine anti-human CD133 antibody (AC133) conjugated to a potent cytotoxic drug, monomethyl auristatin F (MMAF), effectively inhibited the growth of Hep3B hepatocellular and KATO III gastric cancer cells *in vitro* with IC_50_ values of 2–7 ng ml^−1^. MMAF induced apoptosis in the cancer cells as measured by caspase activation. The anti-CD133-drug conjugate (AC133-vcMMAF) was shown to internalise and colocalised with the lysosomal marker CD107a in the sensitive cell lines. In contrast, in the resistant cell line Su.86.86, the conjugate internalised and colocalised with the caveolae marker, Cav-1. Addition of ammonium chloride, an inhibitor of lysosomal trafficking and processing, suppressed the cytotoxic effect of AC133-vcMMAF in both Hep3B and KATO III. Anti-CD133-drug conjugate treatment resulted in significant delay of Hep3B tumour growth in SCID mice. Anti-CD133 antibody-drug conjugates warrant further evaluation as a therapeutic strategy to eradicate CD133+ tumours.

Pancreatic, gastric and hepatocellular cancers are among the most common forms of cancer of the digestive system with an estimated incidence of approximately 38 000, 22 000, and 21 000 cases in the USA for 2008, respectively (Jemal *et al*, 2008). Hepatocellular carcinomas, more common in Asia and sub-Saharan Africa, are increasing in incidence in the USA. Both pancreatic and hepatocellular cancers are fatal diseases, with the number of deaths per year similar to the incidence, and an overall 5-year survival of less than 5% in nonresectable cases. Worldwide, gastric carcinoma is the third most common form of cancer with overall 5-year survival rates of less than 20% as most patients are diagnosed late and are unsuitable for curative surgery. With the challenge of disseminated disease at the time of diagnosis, there is a critical need for finding more effective ways to eradicate the cancer cells.

CD133, an antigenic marker for haematopoietic stem cells (Miraglia *et al*, 1997; Yin *et al*, 1997), is expressed in several haematopoietic malignancies including acute myelogenous leukaemia (Horn *et al*, 1999), acute lymphocytic leukaemia (Buhring *et al*, 1999), chronic lymphocytic leukaemia (Waller *et al*, 1999), and myelodysplastic syndromes (Green *et al*, 2000). Additionally, CD133 expression has been reported in several solid tumours including retinoblastoma (Hemmati *et al*, 2003), glioblastoma (Singh *et al*, 2003, 2004), prostatic adenocarcinoma (Collins *et al*, 2005; Rizzo *et al*, 2005), kidney carcinoma (Florek *et al*, 2005), pancreatic cancer (Hermann *et al*, 2007) and colorectal cancers (O'Brien *et al*, 2007; Van Orden *et al*, submitted). Significantly, CD133-expressing cells in glioblastoma and colorectal cancers include, but are apparently not limited to, the small subpopulation of tumour cells termed cancer stem cells (CSCs) which mediate tumour initiation and metastasis (Singh *et al*, 2004; O'Brien *et al*, 2007; Ricci-Vitiani *et al*, 2007). In addition to being considered the tumour initiating cell population, cancer stem cells have also been demonstrated to be insensitive to chemotherapy and radiation treatment implying that they are responsible for tumour recurrence (Neuzil *et al*, 2007; Tang *et al*, 2007). For example, CD133-positive glioma stem cells have been shown to mediate radiation resistance in highly malignant gliomas (Bao *et al*, 2006). Therapeutic targeting of CSCs populations through a molecule such as CD133 therefore presents a novel opportunity to eradicate tumour initiating, potentially drug-resistant cancer subpopulations.

One goal of this study was to investigate the expression level of CD133 in various solid tumours. Additionally we explored antibody-drug conjugates (ADCs) as a therapeutic modality for the selective targeting of CD133-expressing tumours. Recent advances with ADCs using peptide linkers showed enhanced efficacy and better specificity to antigen-expressing cells (Dubowchik *et al*, 2002; Doronina *et al*, 2003, 2006). Specifically, an anti-human CD133 monoclonal antibody (MAb), AC133, (Yin *et al*, 1997) was directly conjugated to the potent cytotoxic drug, monomethyl auristatin F (MMAF) (Doronina *et al*, 2006), using a valine-citrulline dipeptide linker that is cleavable by proteases (Doronina *et al*, 2003). Our recent study using a panel of normal and cancer tumour lines with varying levels of target antigen demonstrated that the antigen density and trafficking to the lysosomes are important factors for effective killing of the target cells using an ADC (Smith *et al*, 2006). Indeed, we demonstrated that it is possible to kill tumour cells that express antigen at high levels while sparing normal cells expressing the same antigen at a lower level. Here, we demonstrate effective *in vitro* growth inhibition using an anti-CD133 ADC in CD133-expressing hepatocellular carcinoma (Hep3B) and gastric carcinoma (KATO III) cell lines and significant delay of tumour growth *in vivo* for Hep3B xenograft tumours in SCID mice.

## Materials and methods

### Cell lines and culture

Cell lines and the hybridoma AC133. 1 were obtained from the American Type Culture Collection (ATCC, Manassas, VA, USA) and normal human primary cells (HREC, hepatocytes) were obtained from Cambrex (Lonza, Switzerland) and AllCells (Emeryville, CA, USA), respectively. Cell lines were cultured at 37°C with 5% CO_2_ in ATCC-recommended media with 10% fetal bovine serum (FBS) supplemented with 2 mM L-glutamine whereas normal primary cells were grown in media recommended by the suppliers. KATO III was grown in 20% FBS supplemented media. Hybridoma AC133 was grown in hybridoma serum-free media (Invitrogen, Rockville, MD, USA) supplemented with 2.5% FBS and used for purification of MAb, AC133, for *in vitro* and *in vivo* assays.

### Immunohistochemistry

Formalin-fixed paraffin-embedded tissue microarrays were obtained from commercial sources (TriStar, Rockville, MD; USBiomax, Rockville, MD, USA; Imgenex, San Diego, CA, USA; and Petagen/Abxis, Seoul, South Korea). These microarrays include cores containing tumour tissues and corresponding normal tissues. Slides were deparaffinised and processed for antigen retrieval using EZ-retriever system (BioGenex, San Ramon, CA, USA). Samples were preblocked with nonserum protein block (Dako A/S, Glostrup, Denmark) and primary antibodies, used separately, were incubated overnight at room temperature. MAb CD133/1 (AC133) (Miltenyi, Auburn, CA, USA) and control MAb IgG were used at a concentration of 5.0 *μ*g ml^−1^, whereas the anti-CD133 MAb, ab5558 (Abcam, Cambridge, MA, USA), was used at 2.5 *μ*g ml^−1^. Envision Plus system HRP (Dako A/S) was used for detection with 3,3-diaminobenzidine as the substrate for horseradish peroxidase. Slides were then scored using a qualitative scoring scale based on intensity (weak 1+, mild 2+, moderate 3+, strong 4+) and % CD133+ cells (low=<25%, moderate 25–75%, and high >75%). Images were taken using a Zeiss Axiovert 200 M microscope (Carl Zeiss Microimaging, Thornwood, NY, USA). For mouse xenograft tumours, a rabbit anti-CD133 MAb (Cell Signaling Technology, Danvers, MA, USA) and a rabbit polyclonal anti-CD133 (ab19898, Abcam) were used separately as primary antibodies and the Bond Polymer Alkaline Phosphate (AP) Red Detection kit (Vision BioSystems, Australia) for detection.

### Quantitative flow cytometric analysis

Cell surface CD133 expression levels were quantified with QIFIKIT flow cytometric indirect immunofluorescence assay (Dako A/S) using CD133/1 as the primary antibody. A total of 5 × 10^5^ cells per sample were incubated with a saturating concentration (10 *μ*g ml^−1^) of primary antibody for 60 min at 4°C. After washes, FITC-conjugated secondary antibody (1 : 50 dilution) was added for 45 min at 4°C. Binding of antibodies was analysed by flow cytometry and specific antigen density was calculated by subtracting background antibody equivalent from antibody-binding capacity based on a standard curve of log mean fluorescence intensity *vs* log antigen-binding capacity.

### Conjugation of antibodies

MAb AC133 in 50 mM sodium borate, 50 mM NaCl, and 1 mM DTPA pH 8.0 was partially reduced with 2.5 equivalents of Tris(2-carboxyethyl)phosphine hydrochloride at 37°C for 1 h to yield ∼5.3 thiols per antibody. The mixture was cooled to 0°C and partially reoxidised with 0.48 equivalents of 5,5′-dithiobis-(2-nitrobenzoic acid) to ∼4.4 thiols per antibody. This mixture was reacted for 30 min with 1.5 equivalents per thiol of maleimidocaproyl-valine-citrulline-*p*-aminobenzoyl-MMAF (vcMMAF) (Doronina *et al*, 2006). Unreacted drug-linker was quenched with excess *N*-acetyl-cysteine and the mixture purified on a centrifugal S-Fast Flow cation exchange cartridge in 30 mM sodium acetate (pH 5.0) and eluted with PBS. For conjugation to a fluorophore, AC133 or AC133-drug conjugates in 50 mM sodium borate, 50 mM NaCl pH 8.0 were reacted with 6.0 equivalents of Alexa Fluor 488 or 594 *N*-hydroxysuccinimide ester (Invitrogen) at 25°C for 1 h. Conjugates were purified on PD-10 columns equilibrated with PBS.

### Cytotoxicity assay

Cytotoxicity was measured using a resazurin (Sigma, St. Louis, MO, USA) dye reduction assay (McMillian *et al*, 2002). Briefly, cells were plated at 3000 cells per well in 96-well plates, then fresh media was added with or without ADC or AC133 with cross-linking secondary antibody (twofold excess). For inhibition of lysosomal trafficking and processing, Hep3B and KATO III cells were preincubated with 0, 5 or 10 mM ammonium chloride (Sigma, St Louis, MO, USA), a lysosomotrophic agent (Sutherland *et al*, 2006), 30 min prior to incubation with the ADCs. Resazurin was added to cells to a final concentration of 50 *μ*M after 72–96 h of exposure to MAb or ADC. Cells were incubated for 2–6 h, depending on dye conversion of cell lines, and dye reduction was measured on a Fusion HT plate reader (Packard Instruments, Meridien, CT, USA) with excitation and emission wavelengths of 530 and 590 nm, respectively. The IC_50_ value is defined here as the drug concentration that results in 50% reduction in growth or viability as compared with untreated control cultures.

### Cell proliferation assay

Cells were plated, grown and treated as for the cytotoxicity assay in 96 well plates. After 96 h of treatment, 0.5 *μ*Ci well^−1^ of [^3^H]-thymidine (PerkinElmer, 6.7 Ci mmol^−1^) was added to cells and incubated for 6 h at 37°C and 5% CO_2_. To lyse cells, plates were frozen overnight at −20°C and then cell lysates were harvested using FilterMate (Packard Instruments) into 96 well filter plates. Radioactivity associated with cells was measured on the TopCount (Packard Instruments) scintillation counter.

### Measurement of apoptosis

Hep3B cells were plated in 96-well plates at a density of 3000 cells per well in complete media. After 24 h, ADCs were added in increasing concentrations and cells were incubated for 24–72 h. An equal volume of Caspase-Glo 3/7 reagent (Promega, Madison, WI, USA) was added at each time point and plates were read using a Fusion HT plate reader (Packard Instruments, Meridien, CT, USA) after 1 h. Caspase activity of test samples were calculated as a percentage of caspase activity in untreated control cells.

### Immunofluorescence

Cells were grown in coverslip-bottom chamber slides to about 75% confluence. ADC directly conjugated to Alexa Fluor 488 or 594 were added to the cells at 1.0 *μ*g ml^−1^. After 24 h cells were fixed and permeabilised with paraformaldehyde/saponin as provided in the Cytofix/Cytoperm kit (BD Biosciences, San Jose, CA, USA) and then stained with mouse anti-CD107a-FITC (lysosomal marker) (BD Biosciences) or anti-Caveolin-1-Cy5 (Sigma). Nuclei were stained using 4,6-diamidino-2-phenylindole (DAPI, Roche, Switzerland). Cells were mounted with Prolong antifade reagent (Invitrogen). Images were obtained using the Zeiss Axiovert 200 M under oil immersion 63 × objective with apotome for optical sectioning.

### *In vivo* efficacy study

Severe combined immunodeficient mice (SCID, Harlan, Indianapolis, IN, USA) were implanted subcutaneously with 1 × 10^7^ Hep3B cells (ATCC) grown in Minimum Essential Medium Eagle medium (ATCC 30-2003), complemented with of 1% Pen/Strep and 10% FBS. Tumour-bearing mice were randomly divided into groups of seven animals when the mean tumour volume was 100 mm^3^. Mice were then treated by intraperitoneal injection every 4 days for a total of 4 doses with either the anti-CD133 MAb, AC133 at 10 mg kg^−1^, or the corresponding antibody-drug conjugate, AC133-vcMMAF at 1.0 or 3.0 mg kg^−1^, or MOPC21-vcMMAF, at 1.0 or 3.0 mg kg^−1^. MOPC21 (ATCC) was used as nonbinding isotype-matched (IgG1) control MAb to AC133. An additional group of tumour-bearing mice was left untreated as a control. Tumour size was measured two times weekly using calipers. Tumour volume was calculated using the formula, (A × B^2^)/2, where A and B are the largest and second largest perpendicular tumour dimensions, respectively. Animals were euthanised when tumours reached a volume of 1000 mm^3^ or at the end of the study. Tumours were collected for further analysis of CD133 expression by flow cytometry or immunohistochemistry.

For statistical analysis of efficacy data, the log-rank (Mantel–Cox) test was applied using Prism 5.0 (GraphPad Software) to analyse the differences in median tumour quadrupling time between groups. Differences were judged to be significant if *P*⩽0.05. Tumour quadrupling times were determined by nonlinear regression analysis for exponential growth for each experimental animal. Animals that did not reach the quadrupling end point were assigned a quadrupling time as the last day of the study. Actual tumour sizes were used in plotting the data using Prism 5.0. All animal procedures were performed under a protocol approved by the Institutional Animal Care and Use Committee in a facility accredited by the Association for Assessment and Accreditation of Laboratory Animal Care.

## Results

### CD133 is highly expressed in pancreatic, gastric, and hepatocellular carcinomas

**Figure 1:**
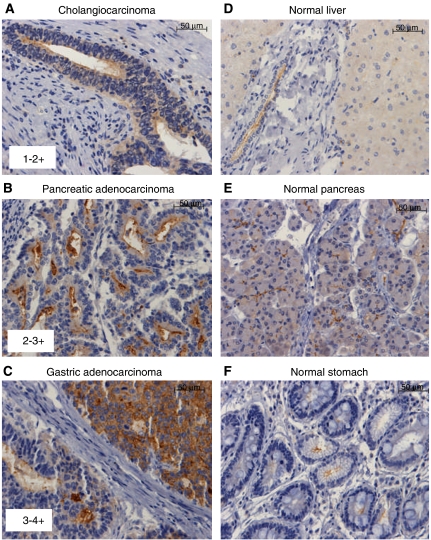
Immunohistochemical analysis of CD133 expression demonstrating the scoring intensity and expression pattern. Representative images using anti-CD133 MAb ab5558: (**A**) liver cholangiocarcinoma with multifocal, minimal to mild membranous and cytoplasmic staining, (**B**) pancreatic adenocarcinoma with mild to moderate membranous (apical) staining of luminal structures, (**C**) gastric adenocarcinoma with moderate to strong staining in two distinct cell populations: (1) luminal and apical and (2) cytoplasmic and membranous, (**D**) normal liver with minimal and nonspecific cytoplasmic staining of hepatocytes and apical staining of bile duct, (**E**) normal pancreas with weak to mild, apical membranous staining of acinar epithelium and ductal epithelium, (**F**) normal stomach with minimal to mild apical staining of glandular crypt epithelium. The scale bars represent 50 *μ*m.

To determine the expression of CD133 in various solid tumours, two MAbs were used separately for the detection of CD133 in formalin-fixed paraffin-embedded samples by immunohistochemistry. An initial survey was conducted with a multi-cancer tissue microarray comprising 12 representative samples from each organ/tissue tumour type: lung, breast, ovary, colon, melanoma, pancreatic, kidney, head and neck, liver, and prostate. Several tumour types were identified with weak to strong staining including hepatocellular carcinomas (12 out of 12 samples) and pancreatic adenocarcinomas (4 out of 12 samples). Additional samples for each tumour type were studied using tissue microarrays specific for pancreatic, gastric, renal, prostatic and hepatocellular cancers. The intensity of the staining for CD133 ranged from weak (1–2+) to strong (3–4+) (Figure 1A–C). Close concordance was observed between the reactivity of both anti-CD133 MAbs in formalin-fixed tissues for most tumour types, with somewhat more variability observed for kidney and prostate cancers (Table 1). The immunostaining pattern was characterised by membranous (apical) as well as luminal staining of the gland-like tumour structures. The apical and luminal pattern of expression is also highly characteristic of the CD133 staining pattern observed in other solid tumours such as colorectal cancers (Figure 1A–C). The nature of the luminal staining is not known, and may represent sloughed cells and/or extracellular membrane particles that contain CD133. However, the staining was CD133 specific, as judged by concordant reactivity between the two anti-CD133 MAbs used and the absence of staining with an isotype-matched negative control MAb.

A high percentage of tumour samples were found to be positive for CD133 in gastric (47–55%), pancreatic (55–68%), and cholangiocarcinomas (67%, biliary type of liver cancer) (Table 1) with the percentages reported based on the analysis using the two MAbs. Qualitative evaluation of the cancer cells within each tumour core showed that gastric carcinomas generally had moderate (25–75%) to high (>75%) percentage of CD133+ while pancreatic and hepatocellular/cholangiocarcinomas cases have generally low (<25%) to moderate % CD133+ tumour cells with some rare high percentage cases. CD133 expression was also detected in 10–38% of renal cell carcinoma cases. Metastatic tumours (20–30 cases primarily of gastric and colorectal origin) included in the gastric and liver tissue microarrays also showed a high percentage (⩾50%) of CD133 positivity, with similar strong membranous and apical pattern of expression (Figure 1). When tumour grades were available, correlation to CD133 immunostaining intensity, distribution or tumour type was evaluated. No significant association with tumour stage or grade was observed for any of the tumour types evaluated with the caveat that a limited number of cases were studied.

CD133 expression in corresponding normal tissues included in the tissue microarrays was also analysed (Figure 1D–F). Weak apical membrane staining was detected in biliary ducts of liver (Figure 1D), pancreatic acinar and ductal epithelium (Figure 1E), and gastric glandular crypt epithelium tubular (Figure 1F). Apical membranous, and, to a lesser degree cytoplasmic, immunostaining was also observed multifocally in normal renal tubular epithelium, glomerular parietal epithelium, and urothelium with each of the anti-CD133 mAbs (data not shown). Thus, CD133 expression levels in these normal tissues are lower than in tumours.

### Anti-CD133 ADCs are potent inhibitors of cell proliferation of Hep3B and KATO III cells

Hepatocellular, pancreatic and gastric cancer cell lines expressing CD133 were identified by quantitative FACS (Table 2). The cell lines used in this study showed a mono-modal flow cytometry profile indicating relatively homogenous expression of CD133. The highest CD133 expression was observed for the hepatocellular cell line, Hep3B with 66000 sites per cell, followed by the pancreatic cell lines Su.86.86 at 36 000 sites per cell and CAPAN-1 at 30 000 sites per cell. The other hepatocellular, pancreatic and gastric cancer cell lines evaluated had lower CD133 expression levels of 6500–12 000 sites per cell. In addition, normal renal epithelial cells and hepatocytes were also tested and displayed minimal to no detectable CD133 expression while we previously determined that CD34-enriched normal bone marrow progenitor cells have <5000 CD133 sites per cell (Van Orden *et al*., 2008).

**Figure 2:**
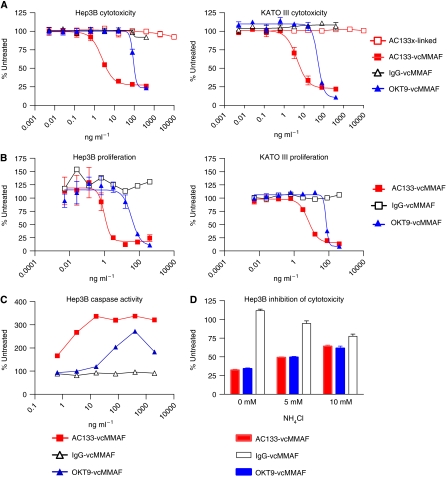
Activity of anti-CD133 ADC against cancer cell lines. (**A**) ADCs targeting CD133 have potent cytotoxic activity against antigen-positive hepatocellular and gastric carcinoma cell lines. Cytotoxicity was measured by resazurin dye conversion in Hep3B and KATO III cells grown in 96-well plates and exposed to anti-CD133 (AC133-vcMMAF) and control ADCs (IgG-vcMMAF and OKT9-vcMMAF) and crosslinked unconjugated anti-CD133 MAb (AC133) for 96 h. (**B**) Proliferation was measured using [^3^H]-thymidine uptake in Hep3B and KATO III cells grown in 96-well plates and exposed to anti-CD133 and control ADCs for 96 h. (**C**) Induction of apoptosis in Hep3B cells treated with AC133-vcMMAF. Caspase 3/7 activation, a quantitative measurement of apoptotic cells, was monitored using the Caspase Glo assay at various time points (24–72 h) after addition of ADCs. Caspase 3/7 activation relative to untreated cells was detected by 48 h with optimal measurement after 72 h in Hep3B cells treated with increasing concentrations of AC133-vcMMAF and positive control OKT9-vcMMAF. (**D**) Inhibition of ADC cytotoxicity using internalisation inhibitor, ammonium chloride (NH_4_Cl) in Hep3B cells. Cells were incubated with increasing concentration of NH_4_Cl 30 min before treated with anti-CD133 (AC133-vcMMAF) or control ADCs. Cytotoxicity was measured after 72 h using the rezasurin dye conversion as in (**A**). The percentage inhibition of cytotoxicity relative to control untreated cells at an ADC concentration of 400 ng ml^−1^ is shown.

Anti-CD133 MAb, AC133 (Yin *et al*, 1997) was conjugated to the anti-tubulin drug, vcMMAF (Doronina *et al*, 2006), with a mean stoichiometry of four drugs per antibody. The resultant ADC, referred to hereafter as AC133-vcMMAF, had potent cytotoxic activity against Hep3B and KATO III cell lines as demonstrated by IC_50_ values ranging from 5–10 ng ml^−1^ in a resazurin dye conversion assay (Figure 2A). In contrast, AC133-vcMMAF had minimal cytotoxic activity (IC_50_ ⩾10 *μ*g ml^−1^) against the other cells tested: HepG2, AGS, Su.86.86, Capan-1 and normal renal epithelial cells and hepatocytes (Table 2). The positive control ADC, OKT9-vcMMAF, targeting the transferrin receptor, was cytotoxic against all cell lines tested (IC_50_ values of 50–100 ng ml^−1^, Figure 2A and B). Unconjugated antibody (AC133) cross-linked to a secondary antibody did not exhibit *in vitro* cytotoxic activity (Figure 2A). When cell proliferation was measured by [^3^H]-thymidine incorporation, potent growth inhibition by AC133-vcMMAF was observed for both Hep3B and KATO III cell lines, with IC_50_ values of 2 and 7 ng ml^−1^, respectively (Figure 2B and Table 2).

To verify that the mode of cell killing by the anti-CD133-drug conjugate is by induction of apoptosis, as observed for other auristatin-containing ADCs (Francisco *et al*, 2003; Law *et al*, 2004, 2006; Sutherland *et al*, 2006), measurement of apoptotic cells were done at several time points after treatment of the cells. The percentage of apoptotic cells relative to untreated cells was measured using a caspase 3/7 activation assay. Apoptotic cells were detected by 48 h with peak caspase activation by 72 h (Figure 2C). Results are shown for Hep3B cells treated with increasing concentration of AC133-vcMMAF and OKT9-vcMMAF, a positive control ADC, and the negative isotype control ADC (mouse IgG-vcMMAF). At 72 h after ADC treatment, maximal caspase activity is observed at 10 ng ml^−1^ concentration of AC133-vcMMAF whereas the positive control OKT9-vcMMAF required a higher concentration (400 ng ml^−1^), concordant with the efficacy of the ADCs as measured by cytotoxicity assay (Figure 2A).

### Subcellular localisation of anti-CD133 ADC in sensitive and resistant cancer cell lines

**Figure 3:**
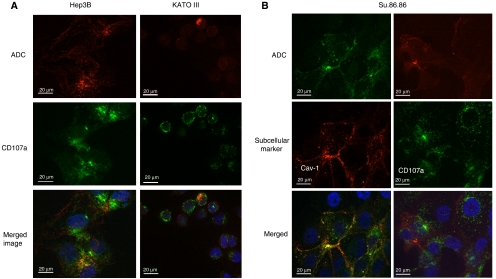
Subcellular localisation of anti-CD133 ADC, AC133-vcMMAF, in sensitive and resistant cancer cell lines. (**A**) AC133-vcMMAF, partially colocalises (yellow) with the lysosomal marker, CD107a, in Hep3B and KATO III cells. Subcellular localisation of AC133-vcMMAF (red) and CD107a (green) in Hep3B and KATO III cells after 24 h incubation with the ADC. (**B**) Subcellular localisation of AC133-vcMMAF, lysosomal marker, CD107a, and caveolin-1 (Cav-1) in Su.86.86 after 24 h incubation with the ADC. AC133-vcMMAF colocalises with Cav-1 (yellow) and not with CD107a in this resistant cell line. Nuclei were stained blue with DAPI. Images were acquired using a × 63 oil immersion objective with Apotome for optical sectioning.

Cells grown in chamber slides were treated with anti-CD133 ADC. A FITC-conjugated anti-CD107a was used to investigate the colocalisation of the ADC (red fluorescence) with the lysosomal marker (green fluorescence) at 24 h (Figure 3A). Lysosomes are rich in proteases such as cathepsin B necessary for effective cleavage and release of the drug from the antibody. Images taken using optical sectioning showed overlap in the fluorescence signal (yellow in the merged image) in Hep3B, indicating colocalisation of the internalised AC133-vcMMAF with the lysosomal marker. Similar colocalisation was seen with KATO III cells, albeit with much lower overall ADC accumulation. In contrast, for the ADC-resistant cell line Su.86.86, colocalisation of the ADC was observed with a caveolae marker Cav-1, but not with the lysosomal marker, CD107a (Figure 3B).

To verify that lysosomal trafficking and processing is required for the ADC efficacy, cytotoxicity experiments were conducted in the presence or absence of ammonium chloride (NH_4_Cl), a lysosomotropic agent that disrupts trafficking and lysosomal processing by neutralising the acidic environment of the endosomal/lysosomal compartments (Fredericksen *et al*, 2002; Sutherland *et al*, 2006). Hep3B cells were preincubated with NH_4_Cl (5 or 10 mM) before addition of 400 ng ml^−1^ anti-CD133 ADC and controls. At this concentration we observed maximal cytotoxicity in Hep3B (Figure 2A). As shown in Figure 2C, addition of NH_4_Cl inhibited cytotoxicity of both AC133-vcMMAF and the positive control ADC (OKT9-vcMMAF) in Hep3B cells. The same protective effect of addition of NH_4_Cl on ADC activity was observed in KATO III cells (data not shown). Dose-dependent effect of NH_4_Cl showed a significant difference between AC133-vcMMAF and negative control ADC (IgG-vcMMAF), although some nonspecific inhibitory effect of higher concentration of NH_4_Cl (10 mM) is observed with the negative control (Figure 2D). These data imply that lysosomal trafficking and processing is one of the important factors for the activity of the anti-CD133-drug conjugate. Other factors, such as intracellular drug concentration and cysteine protease metabolism, impact the overall efficacy of the ADC.

### *In vivo* ADC efficacy study using Hep3B tumours

**Figure 4:**
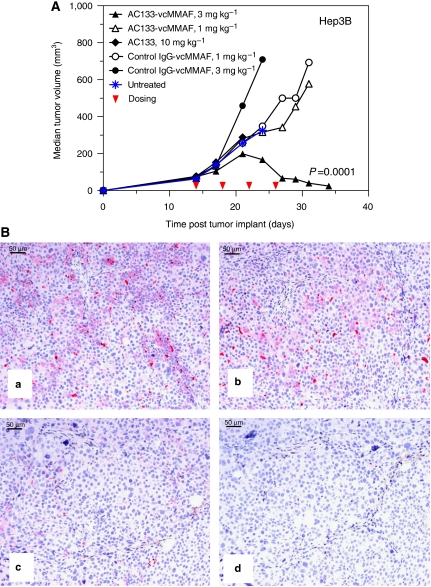
*In vivo* efficacy of an anti-CD133 ADC in Hep3B hepatocellular carcinoma model including IHC analysis of CD133 expression following ADC treatment. (**A**) *In vivo* efficacy of AC133-vcMMAF in Hep3B subcutaneous tumours. SCID mice (*n*=7/group) with established (∼100 mm^3^) Hep3B tumour xenografts were treated by intraperitoneal injection every 4 days for a total of four doses (red arrows) with the anti-CD133 antibody (AC133) or ADC (AC133-vcMMAF) or isotype control mouse IgG1-vcMMAF. An additional group of mice was left untreated as a control. Median tumour volume plots were continued for each group until one or more animals died or were euthanised (see Materials and methods). Tumours were collected when the tumour volume reached 1000 mm^3^. Highly concordant data were in an independent replicate of this experiment. (**B**) CD133 expression in Hep3B xenograft tumours after anti-CD133 drug conjugate treatment. IHC analysis using rabbit anti-CD133 MAb in (**a**) untreated Hep3B xenograft, (**b**) treatment with control IgG-vcMMAF (3.0 mg kg^−1^) and (**c**) treated with AC133-vcMMAF (3.0 mg kg^−1^). (**d**) same tumour as (**c**) stained with control rabbit IgG as primary antibody. Fast Red chromagen was used to detect CD133 expression.

The potent *in vitro* cytotoxic activity of the anti-human CD133 ADC, AC133-vcMMAF, against Hep3B cells prompted us to evaluate the *in vivo* antitumour activity of this ADC against Hep3B xenografts. First, expression of CD133 in Hep3B tumour xenografts was verified by both flow cytometry (not shown) and IHC (Figure 4B). Efficacy experiments were then performed using SCID mice with established (∼100 mm^3^) subcutaneous Hep3B tumours. Mice were treated by intraperitoneal injection with multiple doses (every 4 days for a total of 4 doses) AC133-vcMMAF or an isotype control mouse IgG1-vcMMAF, both as 4-drug loaded ADCS or alternatively with the unconjugated parent MAb, AC133. Mice treated with AC133-vcMMAF at 3.0 mg kg^−1^ showed pronounced anti-tumour response with 2 out of 7 complete responses and 3 out of 7 partial responses and an overall delay in tumour growth (Figure 4A). Tumour volume data were plotted until one or more of the mice in each treatment group (*n*=7) died or was euthanised. Remaining AC133-vcMMAF-treated mice were followed up to day 59 and remaining tumours were collected for analysis of CD133 expression by IHC. Log-rank test of tumour quadrupling time showed a significant difference (*P*=0.0001) in the growth of Hep3B tumours treated with AC133-vcMMAF (3.0 mg kg^−1^) compared to control IgG-vcMMAF at the same dose. In contrast, the naked AC133 antibody (10 mg kg^−1^), AC133-vcMMAF at 1.0 mg kg^−1^ or the control ADC (1.0 or 3.0 mg kg^−1^) exhibited little or no anti-tumour activity. The anti-CD133 ADC, AC133-vcMMAF, was well tolerated at efficacious doses and no overt signs of toxicity were observed. MAb AC133 does not cross-react with murine CD133, thus on-target (antigen-dependent) toxicities were not evaluated in these xenografts experiments.

To determine whether CD133-expressing tumour cells are present in the Hep3B tumours that developed after treatment with AC133-vcMMAF, immunohistochemical analysis was performed using a rabbit MAb that binds to the third extracellular domain of CD133 (Figure 4B) and a rabbit polyclonal Ab generated against a different epitope within the intracellular C terminus (data not shown). Mice that responded with a tumour growth delay following AC133-vcMMAF treatment showed low levels of expression of CD133 within their tumours (Figure 4B-c) indicating either elimination of most CD133+ tumour cells or downregulation of CD133. In contrast, higher levels of CD133 were observed in tumours from mice that were untreated (Figure 4B-a) or treated with a nonbinding control ADC (red stain, Figure 4B-b). Concordant staining was observed with both anti-CD133 rabbit antibodies used, indicating that detection of CD133 in the tumour xenografts was not blocked by ADC treatment.

## Discussion

Our initial observation of CD133 overexpression in colorectal tumours led us to study other tumour types where CD133 may have a potential role in tumour formation. We performed a survey of various tumours and found significant expression of CD133 in pancreatic, gastric, and liver tumours. Metastatic gastric and liver tumours were also found to be CD133-positive indicating that expression is maintained in secondary tumours.

CD133 has been identified as a cancer stem cell marker in brain (Singh *et al*, 2003, 2004), prostate (Collins *et al*, 2005), colorectal (O'Brien *et al*, 2007; Ricci-Vitiani *et al*, 2007) and pancreatic (Hermann *et al*, 2007) cancers. Cancer stem cells are defined as those cells within a tumour that possess the capacity to self-renew and to give rise to the heterogeneous lineages of cancer cells that comprise the tumour (Clarke *et al*, 2006). Strong evidence for the existence of cancer stem cell populations in acute myelogenous leukaemia (Bonnet and Dick, 1997) has invigorated the study of cancer stem cells in both haematologic and solid tumours (Clarke *et al*, 2006). The concept of cancer stem cells implies that these cancer-initiating cells are a critical population that need to be eliminated for cures (Jones *et al*, 2004; Jordan, 2005; Huff *et al*, 2006; Jin *et al*, 2006). On the basis of our studies and other published reports (O'Brien *et al*, 2007; Ricci-Vitiani *et al*, 2007), the proportion of CD133-positive cells is highly variable (up to 25%) across these tumour types. Moreover, not all CD133-positive cells are capable of initiating tumours *in vivo*. Flow cytometry analysis showed that the cell lines studied here have a mono-modal expression of CD133, albeit at different levels for each cell line. For consistent growth of tumours *in vivo*, it required at least 1 × 10^7^ Hep3B cells to be implanted in SCID mice. Thus, CD133 expression is not restricted solely to the cancer stem cells.

In tumour types where CD133 expression is restricted to the putative cancer stem cell subpopulation, tissue microarrays with their limited tumour area for analysis may not provide a representative view of CD133 expression. In hepatocellular, gastric and pancreatic tumours that we analysed using tissue microarrays, we observed a greater percentage of tumour cells that are CD133-positive. As for the cell lines studied, they also strongly suggest that CD133 expression in these tumour types is not limited to the putative cancer stem cells. Larger tissue sections, as compared to tissue microarrays, may increase the likelihood of detecting CD133 expression in cases where the frequency of antigen-positive tumour cells is low. Good concordance using two different anti-CD133 MAbs for the IHC analysis and staining pattern (membranous, apical and luminal) strongly supports the validity of the results obtained here for the expression profiling of CD133.

Apical staining for CD133 is as expected based on the biology of the molecule but the luminal staining in tumours is a novel observation. The nature of the luminal staining for CD133 in primary tumours as well as in the Hep3B *in vivo* model is unknown. Weak, homogeneous staining of luminal necrotic debris and secretory material is often observed in immunohistochemical procedures, and is generally considered nonspecific. In these tumour tissues, however, the luminal immunostaining of sloughed cells and unidentified material was consistently associated with specific staining of tumour cells lining the lumen and of strong intensity that is considered specific. Release of extracellular membrane particles containing CD133 has been reported in neural progenitor cells and some epithelial cells (Marzesco *et al*, 2005). To our knowledge such particles have not yet been reported for tumour cells, although the luminal staining that observed here is consistent with this notion. Using CD133+ tumour cell lines grown *in vitro*, released CD133 was not detected in the culture media. Flow cytometric analysis of dissociated cells from *in vivo* tumours confirmed that CD133 expression is on the cell surface (data not shown). In addition, the efficacy observed *in vivo* demonstrates that CD133 on the tumours is accessible to targeting using an ADC.

CD133 is expressed in normal haematologic, neuronal, and endothelial progenitor cells (Handgretinger *et al*, 2003; Larrivee *et al*, 2005; Shmelkov *et al*, 2005). In a previous study, we determined by quantitative flow cytometric analysis that the level of CD133 in CD34-enriched normal bone marrow progenitor cells is low (<5000 sites per cell) (Van Orden *et al*, submitted). We tested normal CD34+ haematopoietic cells using bone marrow colony formation assay, and as expected because bone marrow progenitor cells are CD133+, colony formation was inhibited by AC133-vcMMAF. The most sensitive cells to the treatment were CFU-GM (IC_50_<0.1 *μ*g ml^−1^) while CFU-GEMM, BFU-E, and CFU-E were more resistant (IC_50_ of 5–10 *μ*g ml^−1^).

We also observed limited apical staining of normal epithelial cells in biliary ducts of the pancreas and gastric glands of low to moderate intensity. Interestingly, the apical nature of CD133 expression in normal human tissues may limit access to antibody and thereby lower the risk of antigen-dependent toxicities. In contrast, cell polarity of CD133 expression is lost for at least poorly differentiated tumours, potentially enhancing the accessibility to antibody targeting (Christiansen and Rajasekaran, 2004; Christiansen *et al*, 2005). Consistent with this notion is the observation that Hep3B cells give rise to poorly differentiated tumours *in vivo* whose growth is slowed by the anti-CD133 ADC, AC133-vcMMAF. Hence, it may be desirable to target CD133+ tumours that are of the poorly differentiated stage rather than well-differentiated tumours with apically expressed CD133.

In previous studies in colorectal cancer, we showed that anti-CD133 ADC can internalise upon binding to CD133 on the cell surface and had cytotoxic and/or anti-proliferative activity against four out of 10 colorectal cancer cell lines tested (Van Orden *et al*, 2008). Immunofluorescence microscopy was used to investigate the internalisation and subcellular localisation of the ADC in both sensitive and resistant cell lines. The ADC is able to efficiently traffic to the lysosome where the linker can be cleaved in sensitive cell lines such as Hep3B and KATO III. Hep3B cells have high CD133 expression (66 000 copies per cell). Moreover, substantial accumulation of the ADC was detected inside the cell within 24 h. Although KATO III cells have more modest levels of CD133 (12 000 copies per cell), colocalisation of the ADC within the lysosome was detected, as well as efficient cell cytotoxicity *in vitro*. In contrast, in Su.86.86 cells which were resistant to the cytotoxic effects of anti-CD133-drug conjugate, the ADC colocalised to caveolae, not the lysosomal compartments. We have previously observed such differential ADC localisation in other sensitive and resistant cell lines with ADCs targeting melanotransferin/p97 (Smith *et al*, 2006). This may provide an additional level of drug specificity to ADCs. CD133 expression in tumours can vary from only a subpopulation of cells, which are putative cancer stem cells, to a significant proportion (>25%) of cancer cells within the tumour, indicating that not all CD133-expressing cancer cells possess stem-like properties. For tumours where CD133 expression is markedly high, using an ADC may prove to be effective in preventing tumour growth. We have shown that the anti-CD133 ADC, AC133-vcMMAF can effectively delay CD133+ Hep3B tumour growth *in vivo*. Targeting CD133+ tumour cells using ADCs has the potential to eliminate CD133+ CSCs as well as antigen-positive tumour bulk.

In addition, due to the presence of non-CD133-expressing cells in primary tumours, to optimise efficacy it may be necessary or desirable to kill adjacent tumour cells that are not expressing CD133. This might potentially be accomplished using ADCs with bystander-killing capability (Kovtun *et al*, 2006). Alternatively, ADCs could be combined with cytotoxic chemotherapy for de-bulking and increasing target accessibility for the ADC. Indeed, clinical benefit from combining an ADC with cytotoxic chemotherapy has recently been demonstrated. Specifically, addition of the ADC, gemtuzumab ozogamicin (Mylotarg) to cytotoxic chemotherapy increased the disease-free survival in a clinical trial in acute myeloid leukaemia (Clavio *et al*, 2007). A recent study of CD133-expressing tumour cells isolated from human glioma xenografts and primary glioblastomas reported that the CD133+ tumour cells may represent the population that confers glioma radiation resistance (Bao *et al*, 2006). These cells preferentially activate the DNA damage check point in response to radiation and effectively repair radiation-induced DNA damage. This implies that radiation therapy in combination with another therapy that effectively targets the CD133+ tumour cells will be required to eradicate the tumour. Anti-CD133 ADCs warrant further evaluation as a therapeutic strategy to eradicate CD133+ tumour cells, including cancer stem cells.

## Figures and Tables

**Table 1:** CD133 expression analysis by immunohistochemistry

		**CD133^+^ cases: ab5558, AC133 (*n*)**			
**Tumour type**	**Total cases (*n*)**	**1–2+**	**2–3+**	**3–4+**	**Total CD133^+^ cases: ab5558, AC133 (%)**
*Primary tumours*					
Gastric adenocarcinomas	60	25, 22	2, 6	6, 0	55, 47
Pancreatic ductal adenocarcinomas	31	13, 15	8, 2	0, 0	68, 55
Kidney and urothelial carcinomas	29	6, 1	5, 2	0, 0	38, 10
Intrahepatic cholangiocarcinomas	12	7, 8	1, 0	0, 0	67, 67
Prostatic adenocarcinomas	39	6, 2	2, 2	0, 0	20, 5
					
*Metastatic tumours*					
Liver^a^	30	7, 10	6, 4	1, 5	47, 63
Gastric	20	6, 6	1, 4	3, 0	50, 50

CD133 expression in solid carcinomas was analysed using tissue microarrays and anti-CD133 MAbs, ab5558 and AC133 and then scored based on the intensity of staining (1–4+, see Figure 1(A–C).

a Predominantly colonic in origin.

**Table 2:** Tumour and normal cell line expression of CD133 and sensitivity to anti-CD133 antibody-drug conjugate, AC133-vcMMAF

**Cell line**	**CD133 copies per cell^a^**	**Cytotoxicity IC_50_ (ng ml^−1^)^b^**	**Growth inhibition IC_50_ (ng ml^−1^)^b^**
*Hepatocellular*			
Hep3B	66 000	5.2±1.0	2.2±0.8
HepG2	10 000	>10 000	>10 000
			
*Gastric*			
KATO III	12 000	9.5	7.0
AGS	6500	10 000	10 000
			
*Pancreatic*			
Su.86.86	36 000	>10 000	>10 000
Capan-1	30 000	>10 000	>10 000
			
*Normal cells*			
Renal epithelial	800	>10 000	>10 000
Hepatocytes	Not detected	>10 000	Not tested

a CD133 expression was estimated by quantitative FACS.

b IC_50_ values (mean±s.e.m.) were calculated as described in Materials and methods from ⩾3 or more independent experiments, except for KATO III (*n*=2).
